# Burden of laboratory-confirmed RSV hospitalization in children <5 years-of-age; 2019–2022

**DOI:** 10.1186/s12887-026-06871-x

**Published:** 2026-04-30

**Authors:** Krow Ampofo, Evan Heller, Alexander Platt-Koch, Per Gesteland, Kaleb Miller, Abbey Loveridge, Lazarus Adua, Yue Zhang, Madelyn Ruggieri, Yoonyoung Choi

**Affiliations:** 1 Department of Pediatrics, University of Utah Health Sciences Center, 81 N Mario Capecchi Drive, Salt Lake City, UT 84113 USA; 2 Department of Internal Medicine, Henry Ford Macomb Hospital, Clinton Township, MI USA; 3 Department of Sociology, University of Utah, Salt Lake City, UT USA; 4 Department of Internal Medicine, Division of Epidemiology, University of Utah, Salt Lake City, UT USA; 5 Value and Implementation Outcomes Research, Merck & Co., Inc, Rahway, NJ USA

**Keywords:** Respiratory syncytial virus, complications, disease severity, hospital cost, early COVID-19 pandemic

## Abstract

**Background:**

Respiratory Syncytial Virus (RSV) is one of the leading causes of acute lower respiratory tract infection (LRTI) in children. We sought to measure the clinical and economic burden of hospitalized RSV LRTI in children.

**Methods:**

We conducted a prospective study of Salt Lake County resident children < 5 years hospitalized with laboratory-confirmed RSV LRTI (hospitalized > 24 h) from 2019 to 2022 at Primary Children’s and Riverton Hospitals, Salt Lake City, Utah. Demographic, clinical, and hospital cost data were abstracted from the electronic medical records and financial transaction database.

**Results:**

Overall, 637 children were hospitalized with laboratory-confirmed RSV infection; 43% <6 months, 36% 6–<24 months, and 21% 24–<60 months years of age. Median hospital LOS was 2.2 days (IQR: 1.6-3.7days) with median and mean hospital cost of $6126 (IQR: $3577-$13436) and $13,941 (SD $26,235) respectively. Admissions to ICU and intubation decreased with age (*p* < 0.01), while antibiotic, steroid, and bronchodilator use increased with age (*p* < 0.01). Hospitalization rates were 24.1, 16.4, 11.1 and 5.5 per 1,000 person-years among children < 6 months, < 1 year, < 2 years and 5 years respectively. Extrapolating to all US children, RSV is estimated to cause ∼101,000 annual hospitalizations nationwide, 63% in otherwise healthy children, with an average total hospital cost of $1.4 billion (95% CI, $683 million–$1.9 billion).

**Conclusions:**

Hospitalized RSV continues to be associated with substantial clinical and cost burden. This underscores the urgent need for widespread implementation of RSV immunoprophylaxis strategies to reduce hospitalization rates.

**Supplementary Information:**

The online version contains supplementary material available at 10.1186/s12887-026-06871-x.

## Background

Respiratory Syncytial virus (RSV) is a leading cause of acute lower respiratory tract infection (LRTI) in children worldwide. It is a common cause of bronchiolitis and pneumonia in young children with the potential to cause severe disease, necessitating hospitalization and intensive care unit (ICU) admissions even in previously healthy children. In the U.S., RSV is estimated to result in 2.1 million outpatient visits, 58,000-100,000 hospitalizations and 100–300 deaths among children < 5 years old [1]. A study estimated RSV-associated hospitalization rates among US infants < 6 months and < 1 year to be 26 (95% CI: 24–28) and 19 (95% CI: 18–21) per 1000 respectively [2]. In other areas with similar climates to the U.S., RSV circulation typically starts during the fall and peaks in the winter and ends in spring, with different regional onset and end dates of seasonal epidemics [3]. 

In late 2019, there was a global pandemic caused by the severe acute respiratory syndrome coronavirus 2 (SARS-CoV-2). The implementation of public health and nonpharmaceutical interventions reduced the transmission of some respiratory viruses including RSV and influenza infection resulting in marked decreases in emergency department and inpatient visits [4]. The clinical and economic burden associated with RSV, influenza virus (IV), parainfluenza virus (PIV), and adenovirus (AV) in children during non-pandemic periods have been reported [5–8]. However, there is currently few data on the clinical and economic impact of children’s hospitalization with RSV infection during the early SARS-CoV-2 pandemic period where the prevalence of respiratory viruses was dynamically changing.

We evaluated the incidence, healthcare resource utilization and costs of hospitalized with RSV LRTI among children < 5 years over a 3-year period (2019 to 2022), while also describing the prevalence of other respiratory viruses.

## Methods

### Study design and setting

We conducted a prospective cohort study among children < 5 years of age residing in Salt Lake County, Utah, who underwent respiratory viral testing for an acute LRTI, including RSV, at Primary Children’s Hospital (PCH) and Riverton Hospital (RH) over a 3-year period (November 2019 through April 2022). PCH is a 289-bed freestanding children’s hospital that serves as both the community hospital for Salt Lake County, UT, and as a tertiary referral center for five states in the intermountain west (Utah, Idaho, Wyoming, Nevada, and Montana). RH is a community hospital for Salt Lake County, UT, with 16 pediatric beds. PCH and RH are owned and operated by Intermountain Healthcare (IH), a not-for-profit, vertically integrated health care system.

Nasopharyngeal (NP) specimens are routinely tested for respiratory viruses in all patients admitted to PCH and RH with respiratory symptoms for the purposes of isolation, cohorting, and medical treatment. Additionally, respiratory viral testing was performed on all febrile infants < 90 days of age. Prior to the SARS-CoV-2 pandemic, respiratory viral testing was conducted by the BioFire^®^ Respiratory Panel (RP) RP2.0 (BioMérieux, Marcy-l’Étoile, France). During the pandemic, testing was conducted by the BioFire FilmArray Respiratory panel RP2.1 which includes the SARS-CoV-2 virus. The indications for respiratory viral testing remained consistent throughout the study period.

### Ethical considerations

The study was conducted in accordance with the principles of the Declaration of Helsinki with ethical approval obtained from the University of Utah and Intermountain Healthcare Institutional Review Board (IRB). Given the retrospective nature of the study, a waiver of informed consent was granted by the IRB under 45 CFR 164.512. Confidentiality and security of patient information were ensured in line with hospital data security protocols.

### RSV outcome measurement

We prospectively identified hospitalizations with laboratory-confirmed RSV LRTI. Two pediatricians (KA, PHG) independently reviewed the principal discharge diagnosis codes based on the International Classification of Diseases, Tenth Revision (ICD-10) for each hospitalization lasting > 24 h. We only included patients with positive test results within three days of admission that also had a physician-diagnosed LRTI using the following ICD-10 admission and discharge codes: bronchitis (J20.x), bronchiolitis (J21.x), and pneumonia (J09.x, J10, J12-18) as well as Acute respiratory failure with hypoxia (J96.x) and Asthma exacerbation (J45.x).

Data abstracted for all eligible patients included hospital length of stay (LOS), the presence of chronic medical conditions (CMC) [9], secondary bacterial infections, intensive care unit (ICU) admission, mechanical ventilation (MV), death, and total hospital costs. To evaluate CMCs that may influence disease severity among children with RSV infection, we examined ICD-10 discharge codes associated with CMCs, as previously described [10, 11]. We assessed MV, secondary bacterial infections, and death as complications. Secondary bacterial infections were defined as the isolation of *Staphylococcus aureus*,* Streptococcus pneumoniae*, typeable *Haemophilus influenzae*,* Moraxella catarrhalis*, or *Streptococcus pyogenes* from a sterile site (blood, pleural fluid, or cerebrospinal fluid [CSF]), or from a respiratory tract specimen if obtained by bronchoalveolar lavage or a protected brush specimen.

The financial transaction database contained within the IH Enterprise Data Warehouse (EDW) includes detailed data about the cost of providing health care. This system identifies and aggregates the variable- and fixed-cost components of patient activities, hospital services, and products according to the date of service [12–14]. Hospital costs incurred in years 2019 to 2022 were standardized to 2023 US dollars by applying a yearly consumer price index adjustment for medical services [15]. 

### Statistical analysis

We compared patient characteristics, resource utilization and hospital cost by season, age, and underlying condition, using Fisher’s exact test and chi square tests (for categorical variables) or Wilcoxon rank-sum test (for continuous variables). Population-based incidence rates were determined for Salt Lake County residents only. The denominator for the incidence of RSV-related hospitalizations was all children < 5 years who were residents of Salt Lake County, which was subsequently stratified by age group using data obtained from the Utah Department of Health’s Indicator-Based Information System for Public Health (IBIS-PH) [16]. The total number of cases identified at PCH and RH was divided by PCH’s and RH’s market share of Salt Lake County hospitalizations for bronchiolitis and pneumonia by age group over the study period (range: 67–71%) to achieve representative incidence rates. To determine national level costs, we multiplied the total population of children < 5 years-of-age in the U.S., obtained from census data [17], by the incidence rate and mean cost reported in this study.

## Results

### Respiratory viral testing and seasons

**Fig. 1:**
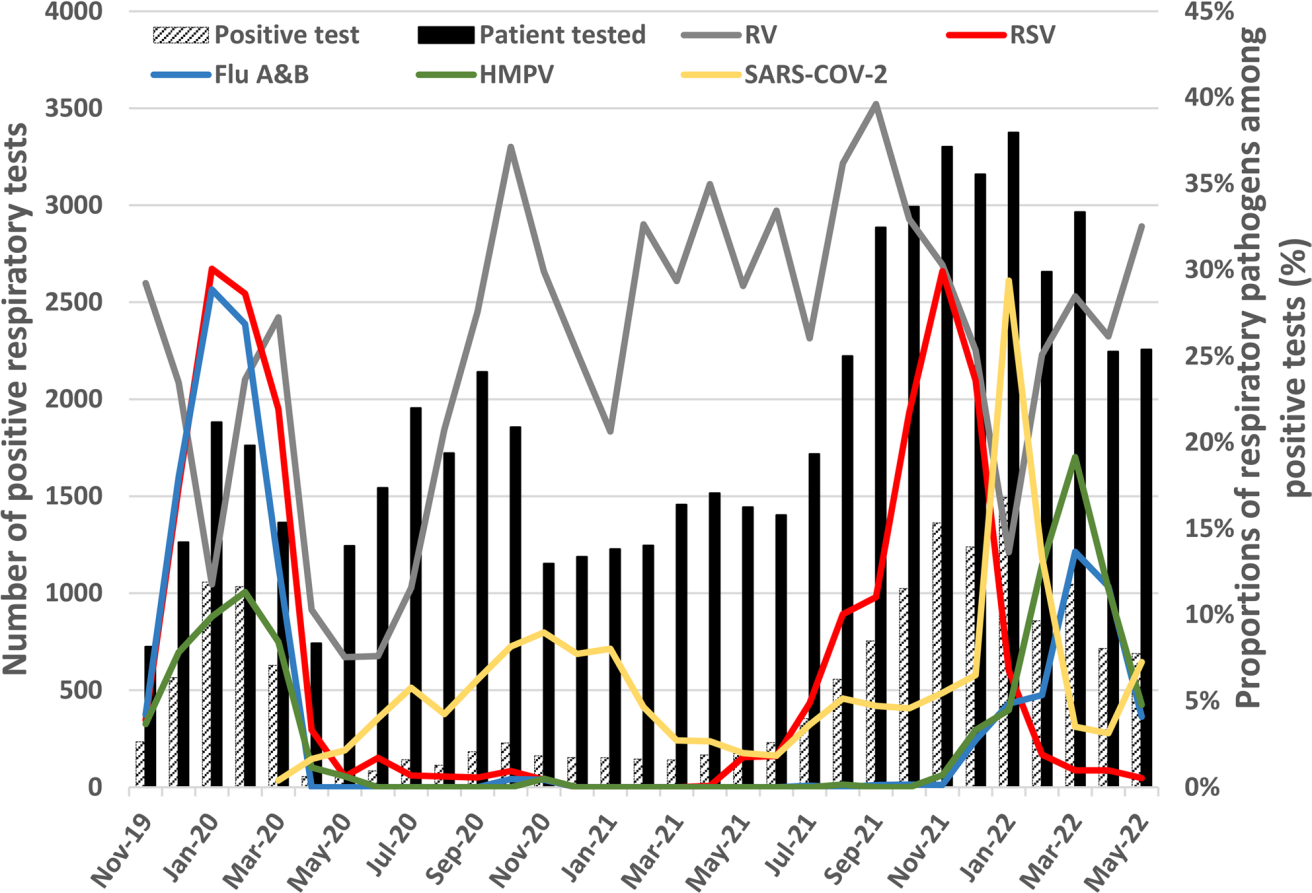
Overall respiratory viral testing among children < 5 years at Primary Children’s Hospital (PCH) and Riverton Hospital (RH), Salt Lake City, Utah from 11/2019 to 5/2022, and the prevalence of respiratory pathogens detected among positive tests. Positive test: positive respiratory viral test for any respiratory virus

Over the study period, 56,328 unique patients < 5 years had a NP sample submitted for respiratory viral testing at PCH and RH (Supplemental Fig. 1). 27% were positive for a respiratory virus; 4423 RV (28%), 4024 RSV (25%), 3371 SARS-CoV-2 (21%), 2193 IV A and B (14%), 1253 AV (8%), 870 HMPV (5.5%), 867 HCoV (5.5%), and 839 PIVs (5.3%). Overall, respiratory viral testing increased during the 2021–2022 winter season compared to previous years (Fig. 1). The proportion of positive viral tests varied over time (4% − 59%) and was lowest during 2020/2021 season. While seasonal occurrences of RSV, IV, and hMPV were minimal during the 2020/2021 season, RV remained constant during the 2020/2021 season.

Of the 4024 RSV test positive children evaluated at PCH and RH, 859 (21%) were hospitalized. There was significant variation in the proportion of children with positive RSV tests who were hospitalized (0% to 30%) over the study period. Over the study period RSV demonstrated winter seasonality for the 2019–2020 and 2021–2022 season (Fig. 1).

### Demographics and clinical characteristics

Of the 859 children with RSV hospitalized at PCH and RH, 637 (74%) were hospitalized > 24 h, were included in the study cohort. We excluded children hospitalized ≤ 24 h as children with mild disease were managed in an observation unit, an extension of the Emergency Department. Five principal ICD-10 codes accounted for 87% of RSV hospitalizations, which included: J21.X – Acute bronchiolitis due to RSV (74%); J96.X – Acute respiratory failure with hypoxia (14%); J12.X – RSV pneumonia (8%); J13 and J15.X – Bacterial pneumonia (2%); and J45.X – Asthma exacerbation (2%) (data not shown).

Most hospitalized children (79%) were < 2 years (Table 1). There was a slight male predominance (55%). One or more CMCs were documented in 158 (25%) children hospitalized with RSV infection. A history of prematurity (birth at < 37 weeks gestation) was the most common, accounting for 14% of CMCs, followed by genetic/metabolic disorders (10%). The proportion of children hospitalized with RSV who had a CMC increased with age (22% among children < 2 years to 36% among children ≥ 2 years, *p* < 0.001) (Table 2). One or more additional respiratory viruses were detected in 30% of children with RSV infection; 43% of co-infections were with RV. Of note, 232 (36%) children received antibiotics, while 148 (23%) were admitted to the ICU, and 36 (6%) required MV.

**Table 1:** Characteristics, resource utilization, and hospital cost among 637 children hospitalized with laboratory-confirmed RSV infection

	All hospitalized(2019-2022) (*n*= 637)	Season 1 (2019-2020)^1^ (*n*=265)	Season 2 (2021-2022)^1^ (*n*=372)	*p*-value
Age (month)				
Mean Median (IQR) <6 months 6-<12 months 12-<24 months 24--<36 months 36-<48 months 48-<60 months	13.6 8.8 (2.5-21.2) 270 (43%) 95 (15%) 135 (21%) 80 (12.5%) 38 (5.5%) 19 (3%)	13.8 9 (2.9-21.2) 103 (39%) 47 (18%) 58 (22%) 37(13.5%) 13 (5%) 7 (2.5%)	13.5 8.3 (2.3-20.7) 167 (45%) 48 (13%) 77 (21%) 43 (11%) 25 (7%) 12 (3%)	NS NS NS NS NS NS NS
Female (%)	288 (45%)	120 (45%)	168 (45%)	NS
Race (%)				
White Pacific Islander/Native Hawaiian Asian Black American Indian/Alaska Native Other	408 (64%) 59 (9%) 14 (2%) 18 (3%) 7 (1%) 129 (20%)	181 (68%) 24 (9%) 7 (3%) 7 (3%) 3 (1%) 43 (16%)	229 (62%) 35 (9%) 7 (2%) 11 (3%) 4 (1%) 86 (23%)	NS NS NS NS NS 0.03
Insurance (%)				
Private IH insurance Medicaid Self-pay	348 (55%) 116 (18%) 274 (43%) 15 (2%)	150 (57%) 53 (20%) 109 (41%) 6 (2%)	198 (53%) 63 (17%) 165 (44%) 9 (2%)	NS NS NS NS
Any chronic medical condition (CMC) (%)^2^	158 (25%)	73 (28%)	86 (23%)	NS
≥ 2 CMC (%) H/o prematurity <37 weeks) (%) Genetic/metabolic (%) Cardiovascular (%) Gastrointestinal (%) Respiratory (%) Neurological/Neuromuscular (%) Renal (%) Hematologic (%) Malignancy (%) Transplant (%)	69 (11%) 86 (14%) 62 (10%) 59 (9%) 29 (5%) 24 (4%) 20 (3%) 18 (3%) 8 (1%) 3 (0.5%) 2 (0.3%)	37 (14%) 37 (14%) 31 (12%) 35 (13%) 14 (5%) 14 (5%) 13 (5%) 7 (2.5%) 4 (1.5%) 1 (0.3%) 1 (0.3%)	32 (9%) 49 (13%) 31 (8%) 24 (6%) 15 (4%) 10 (2.6%) 7 (2%) 11 (3%) 4 (1%) 2 (0.5%) 1 (0.3%)	0.03 NS NS 0.005 NS NS 0.04 NS NS NS NS
RSV alone Co-detection (%)	434 (70%) 203 (30%)	182 (68%) 83 (32%)	252 (68%) 120 (32%)	NS
Culture				
Blood Urine CSF Bronchial protected brush Pleural fluid	136 (21%) 102 (16%) 56 (9%) 13 (2%) 1 (0.2%)	60 (23%) 42 (16%) 25 (9%) 7 (3%)^3^ 0	76 (20%)^4^ 60 (16%)^5^ 31 (8%) 7 (2%)^6^ 1 (0.3%)^7^	NS NS NS NS NS
Any complication	41 (6.4%)	22 (8.3%)	19 (5.1%)	0.13
Mechanical ventilation	36 (6%)	21 (8%)	15 (4%)	0.05
Secondary Bacterial Infection	12 (1.9%)	4 (1.5%)	8 (2.2%)	0.76
Death	1 (0.1%)	1 (0.3%)	-	-
LOS (days)				
Mean Median (IQR)	3.4 2.2 (1.6-3.7)	3.7 2.6 (1.8-3.9)	3.2 2 (1.4-3.6)	0.07
ICU admission (%)	148 (23%)	73 (28%)	75 (20%)	0.04
ICU LOS (days)				
Mean	4.0	4.1	3.9	NS
Median (IQR)	2.7 (1.7-4.5)	1 1.7-5.9	2.5 (1.7-3.7)	
Medication				
IV/oral antibiotics Oral/inhaled steroids Bronchodilators	232 (36%) 120 (19%) 212 (33%)	111 (42%) 54 (20%) 105 (40%)	121 (33%) 66 (18%) 107 (29%)	0.02 NS 0.005
Hospital cost ($)				
Mean SD Median IQR	13,941 26,235 6,126 3,577-13,436	16,773 2,9566 6,146 4,174-14,700	11,920 23,406 5,653 3,240-12,882	0.05

^1^RSV season considered open for palivizumab use when the proportion of RSV test positive is ≥ 5% for two weeks or > 10% in one week and ends when RSV test positive is < 10%. Season 1 was from 11/1/2019 to 4/6/2020. Season 2 was from 8/12/2021 to 4/29/2022

^2^Based of Feudtner classification (Feudtner C, Christakis DA, Connell FA. Pediatric deaths attributable to complex chronic conditions: a population-based study of Washington State, 1980 − 1997. Pediatrics. 2000;106(1 pt 2):205–209)

^3^Bronchial protected brush culture positive (> 10,000 CFU/mL) in 3 patients; *H. influenzae*- 2 and *M. catarrhalis*- 1

^4^Blood culture positive in 2 patients; *E. coli*- 2

^5^Urine culture (catheterized urine specimen with a single pathogen > 50,000 CFU/mL) positive in 4 patients; *E. coli*- 4. Two patients were also culture positive in blood with *E. coli*

^6^Bronchial protected brush culture positive (> 10,000 CFU/mL) in 4 patients; *H. influenzae*- 2, *M. catarrhalis*- 1 and methicillin-sensitive *S. aureus*- 1

^7^Pleural fluid culture positive in 1 patient; *S. pneumoniae*- 1

**Table 2:** Characteristics, resource utilization, and hospital cost among 637 children hospitalized with RSV infection

Age (*n*)	LOS^1^ (d) Mean Median (IQR)	ICU Admission^1^ (%)	Mechanical Ventilation^1^ (%)	Antibiotics^1^ (%)	Steroids^1^ (PO/IH) (%)	Bronchodilator^1^ (%)	Hospital cost^1^ ($2023) Mean (SD) Median (IQR)
<6 m (270) ^2, 3^	3.7 2.3 (1.7-4.1)	84 (31%)	27 (10%)	84 (31%)	20 (7%)	45 (17%)	15,020 (26,344) 6,454 (3,723-15,595)
Healthy (226)	3.4 2.2 (1.7-3.9)	68 (30%)	20 (9%)	70 (31%)	14 (6%)	35 (16%)	13,042 (17,579) 5,837 (3,565-13,892)
CMC (44)	5.1 3.0 (1.9-6.1)	16 (36%)	7 (16%)	14 (32%)	6 (14%)	10 (23%)	25,134 (50,402) 8,952 (4,481-26,313)
6 m to 23 m (230) ^2, 4^	3.4 2.2 (1.5-3.6)	44 (19%)	9 (4%)	89 (39%)	40 (17%)	92 (40%)	15,105 (31,519) 5,837 (3,563-13,111)
Healthy (166)	3.1 2.1 (1.4-3.2)	31 (19%)	7 (4%)	64 (39%)	22 (13%)	58 (35%)	12,343 (29,801) 5,416 (3,315-10,323)
CMC (64)	4.1 2.8 (1.7-4.7)	13 (20%)	2 (3%)	25 (39%)	18 (28%)	34 (53%)	22,340 (34,598) 9,037 (4,466-19,260)
2 y to <5 y (137) ^3, 4^	2.7 2.1 (1.6-3)	20 (15%)	-	59 (43%)	60 (44%)	75 (55%)	9,884 (11,965) 5,794 (3,421-10,174)
Healthy (87)	2.4 2 (1.5-2.9)	11 (13%)	-	36 (41%)	34 (39%)	43 (49)	7,013 (5,558) 5,553 (3,338-8,725)
CMC (50)	3.1 2.2 (1.8-3.3)	9 (18%)	-	23 (46%)	26 (52%)	32 (62%)	14,878 (17,297) 6,851 (4,517-20,534)

d- days, LOS- length of stay, ICU- Intensive care unit, PO- per os, IH- inhaled, IQR- interquartile range, SD- Standard deviation

p-values:

^1^Comparing across age groups: <6 m vs. 6 m to 23 m vs. 2y to <5y: *P* = 0.014 (LOS), *P* = 0.039 (costs), P = < 0.001 (ICU), P = < 0.001 (Mechanical ventilation), *P* = 0.012 (Antibiotics), P = < 0.001 (Steroids), P = < 0.001 (Bronchodilator)

^2^Comparing age groups <6 m vs. 6 m to 23 m: *P* = 0.07 (LOS), *P* = 0.18 (costs), *P* = 0.002 (ICU), *P* = 0.009 (Mechanical ventilation), *P* = 0.08 (Antibiotics), P = < 0.001 (Steroids), P = < 0.001 (Bronchodilator)

^3^Comparing age groups <6 m vs. 2y to <5y: *P* − 0.022 (LOS), *P* − 0.049 (costs), P = < 0.001 (ICU), P = < 0.001 (Mechanical ventilation), *P* = 0.017 (Antibiotics), P = < 0.001 (Steroids), P = < 0.001 (Bronchodilator)

^4^Comparing age groups 6 m to 23 m vs. 2y to <5y: *P* = 0.46 (LOS), *P* = 0.42 (costs), *P* = 0.27 (ICU), *P* = 0.03 (Mechanical ventilation), *P* = 0.41 (Antibiotics), P = < 0.001 (Steroids), *P* = 0.006 (Bronchodilator)

### Population-based incidence of RSV LRTI hospitalization

**Fig. 2:**
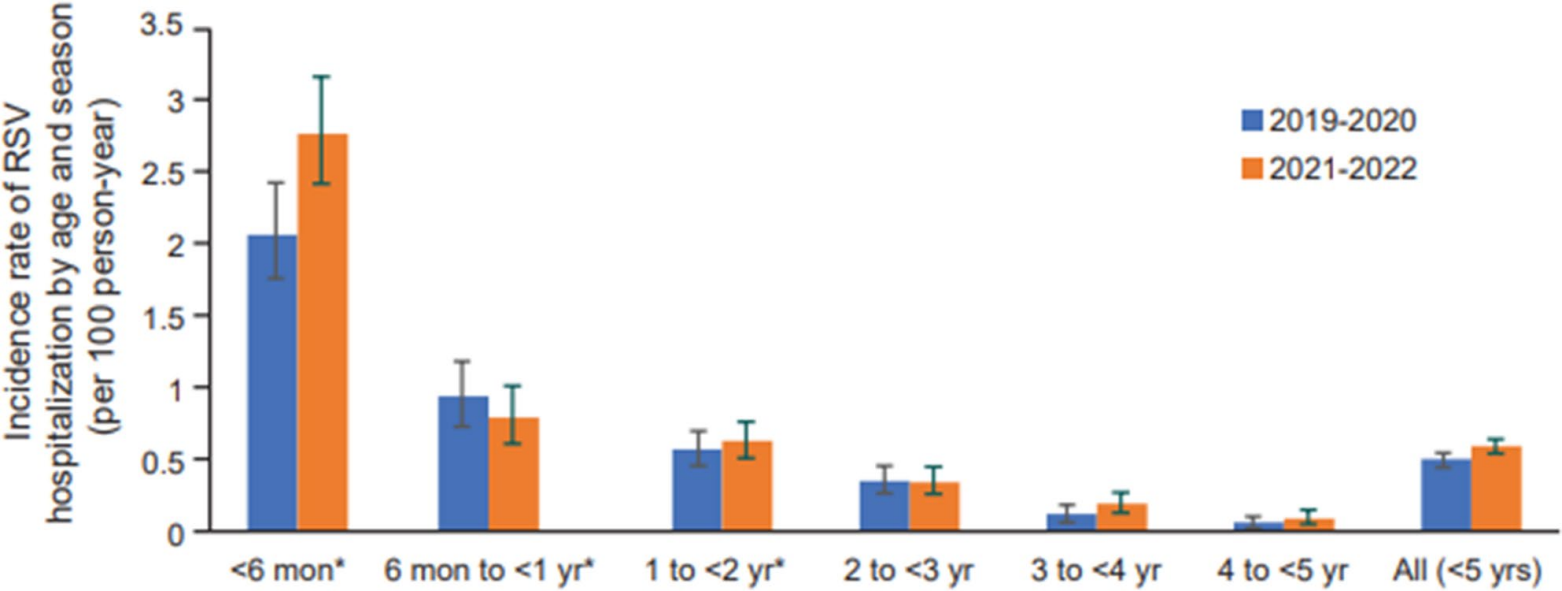
Population-based incidence of RSV hospitalization per 100 person-year by season

The mean RSV hospitalization rate for Salt Lake County residents is shown in Fig. 2 and Supplemental Table 1 A and 1B. The overall mean hospitalization rate over the 2 seasons with community RSV activity was 0.55 per 100 person-years among children < 5 years; 1.1 per 100 person-years among children < 2 years and 1.64 per 100 person-years for children < 1 year of age (data not shown). The mean hospitalization rates were highest in children < 6 months (2.41 per 100 person-years) and dropped sharply with increasing age.

**Fig. 3:**
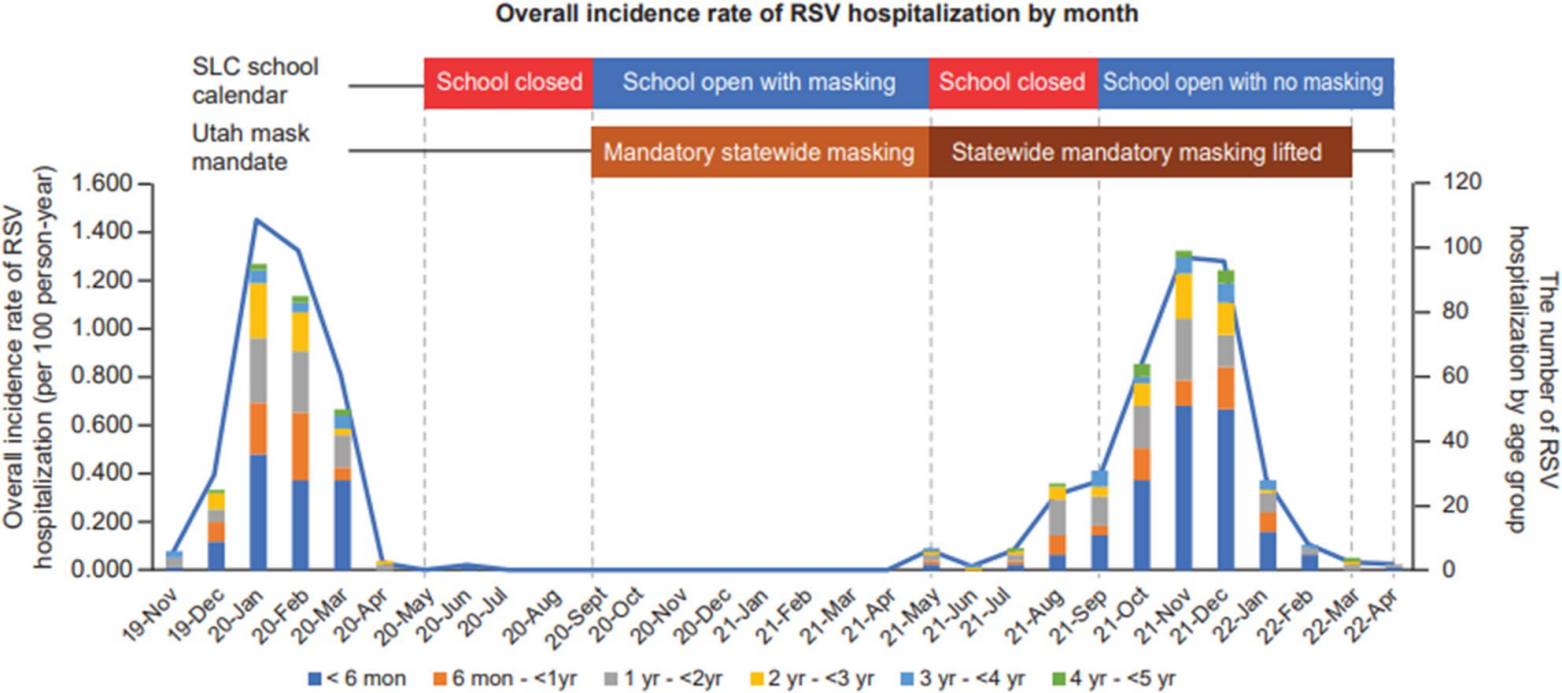
Monthly incidence and number of RSV hospitalizations with local public health preventive measures. Note: The primary y-axis corresponds to the line graph, while the secondary y-axis corresponds to the stacked bar graphs

There was no RSV activity and hospitalization during the 2020–2021 winter season, which coincided with a variety of public health measures, including school closures and statewide mandated masking in Utah (Fig. 3). When RSV was circulating, hospitalization incidence peaked in January during the 2019–2020 season and in November during the 2021–2022 season. IV and hMPV hospitalization mirrored the observed RSV activity.

### Hospital resource utilization and cost

Overall, the median hospital LOS was 2.2 days (IQR: 1.6-3.7days), the median cost was $6126 (IQR: $3577-$13436), and mean cost was $13,941 (SD $26,235) (Table 1). There were significant decreases in hospital LOS, hospital cost, ICU admissions, and MV with increasing age, while antibiotic, steroids and bronchodilator use increased with increasing age. By age group, hospital LOS and cost among children < 6 months of age were comparable to those 6 months to 23 months, but significantly higher than children > 2 years. Resource utilization and therapeutic interventions were higher among children < 6 months compared to those 6 months to 23 months and children > 2 years (Table 2).

Compared with healthy children, those with at least one CMC had significantly longer LOS (median LOS: 2.5 days; IQR 1.8–4.8 days vs. 2.1 days; IQR 1.6–3.6 days; *p* = 0.004) and higher costs (median cost: $8721, IQR: $4521–20783 vs. $5691, IQR: $3403-$11542; *p* = 0.0002) across all age groups. Children with two or more CMCs had similar hospital LOS (median LOS: 2.4 days; IQR 1.9–5.4 days vs. 2.7 days; IQR 1.7–4.6 days; *p* = 0.5) and hospital costs (median cost: $11008, IQR: $5473–30739 vs. $7492, IQR: $4172-$15689; *p* = 0.06) when compared to those with one CMC (data not shown).

Overall, hospital costs for ICU admissions were significantly higher than non-ICU admissions (median cost: $20623, IQR: $14092-$35850 vs. $4886, IQR: $3281-$8055; *p* < 0.0001), and similar among all age groups; <6 months (median cost: $23558, IQR: $15568-$39379 vs. $5108, IQR: $3235-$7590; *p* < 0.0001), 6 to 23 months (median cost: $18345, IQR: $13828-$36732 vs. $5905, IQR: $3303-$8213; *p* < 0.0001), and 2 to < 5 years (median cost: $9367, IQR: $10074 -$23006 vs. $5712, IQR: $3291-$8429; *p* < 0.0001) (data not shown).

The total RSV hospitalization cost in our study varied annually when RSV was in circulation, ranging from $4.4–4.5 million per season, with a total direct cost of $8.9 million over the study period (data not shown). Children < 6 months, 6 months -<1 year, 1-<2 years and 2-<5 years accounted for 46%, 21%, 18%, and 15% of the total hospital cost respectively. Healthy children accounted for the majority (63%) of total hospital costs over the study period (data not shown). If extrapolated to the US population < 5 years of age, we estimate that RSV may result in ~ 101 000 hospitalizations annually in children < 5 years, with a mean estimated hospital cost of $1.4 billion (95% CI, $683 million–$1.9 billion) (data not shown) [17]. 

### Complications

Over the study period, 41 patients (6.4%) experienced complications, including MV, secondary bacterial infections, and death (Table 1). Respiratory failure necessitating MV occurred in 36 children (6%), with a trend of fewer cases during season 2 compared to season 1 (4% vs. 8%; *p* = 0.05). A secondary bacterial infection was observed in 12 children (7.2%) who had specimens obtained for bacterial culture (1.9% of all hospitalized children with RSV). The most common bacteria isolated was *E. coli* (33%), all from children with concurrent UTI, while *H. influenzae* (50%), *M. catarrhalis* (25%), *S. aureus* (12.5%) and *S. pneumoniae* (12.5%) were isolated from deep lower respiratory samples and pleural fluid from 8 children with pneumonia with or without an effusion. No bacteria were isolated from the CSF samples submitted for culture.

There was one death in an infant with congenital hypotonia hospitalized with RSV bronchiolitis, necessitating intubation and MV, and venoarterial extracorporeal membrane oxygenation (VA-ECMO) due to cardio-respiratory failure. At the time of death, admission blood cultures and tests for other viral pathogens were negative. The extent to which RSV contributed to the death is unknown.

## Discussion

In this study of children hospitalized with laboratory-confirmed RSV between pre- and post-periods of the SARS-CoV-2 pandemic, there were significant changes in the epidemiology of community-associated RSV. This study period immediately pre-dated the availability of preventive measures for RSV including maternal immunization and monoclonal antibodies. There was substantial clinical and economic burden of RSV infection as demonstrated by hospitalization rates, healthcare resource use, and hospital costs. First, there was a change in RSV seasonality with no RSV activity during the 2020–2021 winter season that coincided with a variety of public health measures limiting transmission of respiratory viruses. Secondly, the mean incidence of RSV hospitalization among Salt Lake County residents between 2019 and 2022 RSV seasons was 0.55 per 100 person-years among children < 5 years and 1.11 per 100 person-years among children < 2 years, with the highest in children < 6 months (2.41 per 100 person-years). Thirdly, severe disease, manifest as ICU admission and intubation with MV decreased with increasing age (*p* < 0.01), while antibiotic, steroid, and bronchodilator use increased with age (*p* < 0.01). Lastly, extrapolating our data, we estimated that RSV caused about 101,000 annual hospitalizations in U.S. children under 5, resulting in mean hospital costs of approximately $1.4 billion (95% CI: $683 million–$1.9 billion).

Over the study period, RSV was the second most common respiratory virus detected in respiratory samples tested in children < 5 years after rhinovirus/enterovirus, accounting for 7% of all respiratory viral tests and 27% of all the respiratory viruses detected. In previous studies, RSV was typically the most common respiratory virus detected in children presenting to emergency room with bronchiolitis [18] as well as among those hospitalized with community-acquired pneumonia [19]. These differences may be due to the increased and sustained respiratory viral testing associated with any healthcare encounter during the early stages of the SARS-CoV-2 pandemic [20]. Our findings support previous reports and the importance of RSV as a cause of respiratory infections in children [21]. 

Earlier reports have described changes in the pattern and epidemiology RSV disease since the SARS-CoV-2 pandemic. In our study, there was no change in the median age of hospitalized children during the 2109 − 2020 and 2021–2022 seasons. Our findings are consistent with previously published reports by Agha et al. and McMorrow et al. [22, 23], but contrast with other reports that describe RSV infection occurring in older children in the post-SARS-CoV-2 pandemic [24–27]. Differences in the reported ages may be due to time periods studied. The virtual absence of RSV disease during the 2020–2021 season is similar to what has been reported elsewhere. It likely reflects the ability of social isolation, mask use and closure of pre-schools and elementary schools to disrupt transmission. Viral interference from SARS-CoV-2 has also been postulated, but the re-appearance of RSV disease in 2021–2022 during the omicron surge might argue against interference playing a large role.

Previous studies have reported variable markers of disease severity in children with RSV, before and after the SARS-CoV-2 pandemic. In our study, compared to the 2019–2020 season, fewer children received ICU care, underwent intubation and MV, received antibiotics or were treated with bronchodilator therapy, with no change in hospital LOS during 2021–2022 season, suggesting less severe RSV disease in hospitalized children [28, 29]. Our findings are comparable to previous studies that demonstrated variability in healthcare resource use during the early SARS-CoV-2 pandemic [22–24, 30–32]. In this study, there was one death (0.6%), which is consistent with previous reports describing the low case-fatality rate associated with pediatric RSV infections [28, 29, 33], despite the large overall burden and the proportion of children (~ 25%) with CMCs [23, 28, 29, 33–35]. The proportion of RSV hospitalized children with secondary bacterial infections in our study (1.9%) is comparable to that previously reported for other common respiratory viruses: HPMV (2.2%) [8], influenza (< 2%) [5, 36], RSV (~ 1%) [37], or AV (1.4%) [38]. Interactions between respiratory viruses and subsequent bacterial infections, especially secondary bacterial pneumonia, have been well documented [39]. 

The RSV hospitalization rates in our study were comparable to the Centers for Disease Control and Prevention’s New Vaccine Surveillance Network (NVSN) of 4.0, 5.2 and 6.0 per 1,000 children < 5 years during the 2016–2020, 2021 and 2022 seasons respectively [23, 34]. Similar to our study, the NVSN is population-based and tests all hospitalized children. The hospitalization rates observed in our study are higher compared to influenza during the same time study period, which reported rates of 66.8 to 91.5 per 100,000 children under 5 years old, based on hospitalization surveillance networks [40]. 

Using a cost-based system we found that the median cost of an RSV hospitalization was $6126 (IQR: $3577-$13436), and mean cost was $13,941 (SD $26,235); charges would be substantially higher. Extrapolating for our data, we estimate that the costs of RSV hospitalization for children under five years are at least $1.4 billion (95% CI, $683 million–$1.9 billion) based on 101,000 RSV hospitalizations per year. Cost of care in Utah is lower than for many regions so this estimate may be conservative. The overall estimate is comparable to the 106,000 hospitalizations reported by McLaughlin et al., who used data from pooled RSV studies [2]. Additionally, our cost estimates are similar to the $709.6 million reported for infants with RSV hospitalization, which estimated costs based on a systematic literature review [41]. 

Our study has limitations. First, despite prospective outcome ascertainment through active surveillance, we abstracted data from the institutions’ EDW and financial transaction database, the completeness and accuracy of which were not verified manually. Second, this study focused on children hospitalized > 24 h to capture moderate to severe disease and may have underestimated RSV hospitalization and associated costs. Third, although we reviewed outliers for hospital costs and LOS and excluded patients whose primary reason for admission was not attributable to RSV infection, not all outcomes are necessarily due entirely to RSV. Fourth, our estimates of the economic burden of RSV hospitalizations do not account for indirect medical costs such as parental work absences. Lastly, this study was conducted in a single region and finding and results may not be generalizable to all hospitalized children across the United States. Similarly, extrapolated national-level costs should be interpreted with caution, as it may understate or overstate the true economic burden.

## Conclusion

In conclusion, RSV was a significant respiratory pathogen in the pre- and post- SARS-CoV-2 pandemic periods, leading to considerable hospital admissions and financial burden, particularly among infants and young children. While the rates of ICU admissions and mechanical ventilation decrease with age, treatment interventions such as antibiotics, steroids, and bronchodilators increase with age. National RSV hospitalization costs exceed $1.4 billion annually, with a substantial portion attributable to otherwise healthy children. These insights highlight the urgent need for the widespread implementation of RSV immunoprophylaxis strategies to reduce RSV hospitalization rates.

## Supplementary Information

Supplementary Material 1.

## Data Availability

The Study data that support the findings of this study are not publicly available due to the data containing protected health information. All inquiries about access to the data should be sent to the corresponding author.

## Abbreviations

PCH: Primary Children’s Hospital
RH: Riverton Hospital
IH: Intermountain Healthcare
EDW: Enterprise Data Warehouse
ICD-10: International Classification of Diseases, 10th Revision
ICU: Intensive care unit
RSV: Respiratory syncytial virus
RV: Rhinovirus
HMPV: Human metapneumovirus
PIV: Parainfluenza virus
IV: Influenza virus
AV: Adenovirus
SARS-CoV-2: Severe acute respiratory syndrome coronavirus 2
HCoV: Human coronavirus
IQR: Interquartile range
SD: Standard deviation
LOS: Length of stay
LRTI: Lower respiratory tract infection
CMC: Chronic medical conditions
