# Supplementary material for: Burden of laboratory-confirmed RSV hospitalization in children <5 years-of-age; 2019–2022
Source: BMC Pediatr. 2026 Apr 30;26:706. doi: 10.1186/s12887-026-06871-x (PMC13440060; doi:10.1186/s12887-026-06871-x)
Supplement: Supplementary file 1 [file 12887_2026_6871_MOESM1_ESM.docx]

**Supplemental Figure 1: Method of Respiratory Syncytial Virus (RSV) hospitalization ascertainment among children <5 years evaluated at Primary Children’s Hospital (PCH) and Riverton Hospital (RH), Salt Lake City, Utah, from 11/2019 to 5/2022.**

**Excluded**	3165 RSV+ discharged home from PCH and RH ER

4204 test RSV+ at PCH and RH

56328 children <5 years had a respiratory sample submitted for respiratory viral testing at PCH and RH

**Excluded**	4423 RV+	3371 S-CoV-2+	2193 IV A/B+	1253 AV+	870 HMPV+	867 HCoV+	839 PIVs+	38488 test negative

222 RSV+ hospitalized ≤24 hours at PCH and RH observation unit	(not analyzed)

859 RSV+ hospitalized at PCH and RH

**Study Population**

**637 RSV+ hospitalized >24 hours at PCH and RH (Analyzed)**

PCH- Primary Children’s Hospital, RH- Riverton Hospital, ER- Emergency room, RSV- Respiratory syncytial virus**,** RV- Rhinovirus, S-CoV-2- Severe acute respiratory syndrome coronavirus-2, IV A/B- Influenza virus A/B, AV- Adenovirus, HMPV- Human metapneumovirus, HCoV-Human Coronavirus, PIV- Parainfluenza Virus.

**Supplemental Table 1A: Population-Based Incidence of RSV Hospitalization per 100 person-year (2019-2020)**

| **Age** | **2019**  **Salt Lake County  Population** | **Uncorrected** | | **Corrected for Market share^1^** | |
| --- | --- | --- | --- | --- | --- |
|  |  | **Number hospitalized** | **Hospitalization /100 person year (95% CI)** | **Number hospitalized** | **Hospitalization /100 person year (95% CI)** |
| **< 6 mon^2^** | 7480 | 103 | 1.38 (1.13-1.67) | 154 | 2.06 (1.75-2.41) |
| **6 mon to <1 yr^2^** | 7480 | 47 | 0.63 (0.46-0.83) | 70 | 0.94 (0.73-1.18) |
| **1 to <2 yr*** | 15281 | 58 | 0.38 (0.29-0.49) | 87 | 0.57 (0.46-0.7) |
| **2 to <3 yr** | 15529 | 37 | 0.24 (0.17-0.33) | 55 | 0.35 (0.27-0.46) |
| **3 to <4 yr** | 15944 | 13 | 0.08 (0.04-0.14) | 19 | 0.12 (0.07-0.19) |
| **4 to <5 yr** | 16908 | 7 | 0.04 (0.02-0.09) | 10 | 0.06 (0.03-0.11) |
| **All (< 5 yrs)** | 78621 | 265 | 0.34 (0.3-0.38) | 395 | 0.5 (0.45-0.55) |

^1^Riverton hospital and Primary Children’s Hospital market share for Salt Lake County children hospitalized with RSV for >24 hours (265/397) 67%

^2^ Assumes 1 year of age is divided almost equally between 6 months and 6 months to 1 year.

**Supplemental Table 1B: Population-Based Incidence of RSV Hospitalization per 100 person-year (2021-2022)**

| **Age** | **2021**  **Salt Lake County  Population** | **Uncorrected** | | **Corrected for Market share^1^** | |
| --- | --- | --- | --- | --- | --- |
|  |  | **Number hospitalized** | **Hospitalization /100 person year (95% CI)** | **Number hospitalized** | **Hospitalization /100 person year (95% CI)** |
| **< 6 mon^2^** | 7860 | 167 | 2.12 (1.82-2.47) | 217 | 2.76 (2.41-3.15) |
| **6 mon to <1 yr^2^** | 7860 | 48 | 0.61 (0.45-0.81) | 62 | 0.79 (0.61-1.01) |
| **1 to <2 yr*** | 15888 | 77 | 0.48 (0.38-0.6) | 100 | 0.63 (0.51-0.76) |
| **2 to <3 yr** | 16260 | 43 | 0.26 (0.19-0.36) | 56 | 0.34 (0.26-0.45) |
| **3 to <4 yr** | 17101 | 25 | 0.15 (0.09-0.22) | 33 | 0.19 (0.13-0.27) |
| **4 to <5 yr** | 17287 | 12 | 0.07 (0.04-0.12) | 16 | 0.09 (0.05-0.15) |
| **All** | 82256 | 372 | 0.45(0.41-0.5) | 483 | 0.59 (0.54-0.64) |

^1^Riverton hospital and Primary Children’s Hospital market share for Salt Lake County children hospitalized with RSV for >24 hours (370/483) 77%

^2^Assumes 1 year of age is divided almost equally between 6 months and 6 months to 1 year.
